# N-Acetylcysteine and Atherosclerosis: Promises and Challenges

**DOI:** 10.3390/antiox12122073

**Published:** 2023-12-04

**Authors:** Yuqi Cui, Qiang Zhu, Hong Hao, Gregory C. Flaker, Zhenguo Liu

**Affiliations:** 1 Department of Geriatrics, Donald W. Reynolds Institute on Aging, University of Arkansas for Medical Sciences, 4301 West Markham, Little Rock, AR 72205, USA; ycui@uams.edu; 2 Center for Precision Medicine and Division of Cardiovascular Medicine, Department of Medicine, School of Medicine, University of Missouri, Columbia, MO 65212, USA

**Keywords:** atherosclerosis, N-acetylcysteine, inflammation, oxidative stress, antioxidant

## Abstract

Atherosclerosis remains a leading cause of cardiovascular diseases. Although the mechanism for atherosclerosis is complex and has not been fully understood, inflammation and oxidative stress play a critical role in the development and progression of atherosclerosis. N-acetylcysteine (NAC) has been used as a mucolytic agent and an antidote for acetaminophen overdose with a well-established safety profile. NAC has antioxidant and anti-inflammatory effects through multiple mechanisms, including an increase in the intracellular glutathione level and an attenuation of the nuclear factor kappa-B mediated production of inflammatory cytokines like tumor necrosis factor-alpha and interleukins. Numerous animal studies have demonstrated that NAC significantly decreases the development and progression of atherosclerosis. However, the data on the outcomes of clinical studies in patients with atherosclerosis have been limited and inconsistent. The purpose of this review is to summarize the data on the effect of NAC on atherosclerosis from both pre-clinical and clinical studies and discuss the potential mechanisms of action of NAC on atherosclerosis, as well as challenges in the field.

## 1. Introduction

Atherosclerosis remains a leading cause of cardiovascular diseases (CVDs) globally and is considered a chronic inflammatory disease, with increased levels of reactive oxygen species (ROS) and oxidative stress [1,2]. Antioxidants, which inhibit oxidation, would be expected to have a favorable impact on patients with atherosclerosis. However, the Heart Outcomes Prevention Evaluation (HOPE) Study [3], a double-blind and randomized trial with patients at high risk for cardiovascular events, showed that treatment with antioxidant vitamin E had no beneficial effect over a mean follow-up of 4.5 years. Although there were no significant adverse effects of vitamin E, the primary outcome (a composite of myocardial infarction, stroke, and death from cardiovascular causes) and the secondary outcomes (including unstable angina, congestive heart failure, revascularization or amputation, death from any cause, complications of diabetes, and cancer) were the same in patients either on vitamin E or placebo [3]. Studies with antioxidant β-carotene treatment also failed to achieve significant clinical benefits in patients with CVDs, including atherosclerosis [4].

N-Acetylcysteine (NAC) is approved by the Food and Drug Administration (FDA) for the treatment of acetaminophen overdose. Although not approved for use as a dietary supplement, NAC has been widely used for acute respiratory distress syndrome, bronchitis, chemotherapy-induced toxicity, human immunodeficiency virus/acquired immune deficiency syndrome, radio-contrast-induced nephropathy, heavy metal toxicity, psychiatric disorders, and as an over-the-counter nutritional supplement [5,6]. In the cardiovascular area, NAC has been used off label for doxorubicin-induced cardiotoxicity, stable angina pectoris, and cardiac ischemia-reperfusion injury [6,7].

The primary mechanisms for the actions of NAC are considered to relate to its antioxidative effects via increasing intracellular glutathione (GSH) levels (crucial for cellular redox balance) and its anti-inflammatory effect through suppressing nuclear factor kappa B (NF-κB)-mediated expression of a variety of inflammatory mediators, including tumor necrosis factor-alpha (TNF-α) and interleukins (IL-6 and IL-1β) [5]. In this review, we summarize the data on the effects of NAC on atherosclerosis from both pre-clinical and clinical studies and discuss the potential mechanisms of the effects of NAC on atherosclerosis development and progression, as well as controversies and challenges concerning NAC and atherosclerosis. The actions and mechanisms of NAC on the modulation of lipid metabolism, homocysteine, and vascular endothelial cells will also be discussed.

## 2. Overview of NAC and Cardiovascular Diseases

As shown in Table 1, the intravenous administration of NAC significantly increases arterial vascular reactivity during reactive hyperemia in patients with chronic kidney disease following hemodialysis [8], reduces vasospasm in patients suffering from subarachnoid hemorrhage [9], and prevents ischemia-reperfusion syndrome following aortic clamping in patients during abdominal aortic aneurysmectomy [10]. NAC has been shown to decrease the frequency and severity of Raynaud’s phenomenon (RP) attacks and digital ulcers (DU) in patients with systemic sclerosis (SSc), with a significant reduction in plasma adrenomedullin concentrations [11,12,13]. Another study also demonstrated that NAC protected patients with RP secondary to SSc against DU, although NAC has no significant vasodilator effect on the microcirculation in hands [14].

NAC has been reported to protect against coronary artery diseases (CAD), myocardial infarction (AMI), myocardial injuries, and cardiomyopathy [15,16,17,18,19,20] (Table 2). In patients with cardiac surgery, NAC decreases diabetes-associated cardiovascular, cardiopulmonary bypass, and cardiac surgery complications, including early reperfusion injury, pump-induced inflammatory response, and myocardial stress [21,22,23]. However, a systematic review has shown that NAC has no significant efficacy in improving major adverse outcomes, including mortality, acute renal failure, HF, length of stay and/or outcomes of care in intensive care unit, arrhythmia, and AMI, in patients following cardiac surgery [24]. Multiple clinical studies have demonstrated that NAC treatment effectively reduces the risk of atrial fibrillation (AF) (Table 3), a common arrhythmia post cardiac surgery [25,26,27,28,29], although one study reveals that a high dose of oral NAC treatment had no benefit on postoperative AF [30]. In addition, NAC has been reported to improve HF [31] however, no beneficial effect was observed in patients with doxorubicin-induced cardiomyopathy [32,33].

Animal studies have shown that NAC improves peripheral vascular diseases (PVD), with reduced muscular fatigue [40], restoration of redox balance and calcium retention capacity, as well as the suppression of reactive oxygen species (ROS) production in mice with hind limb ischemia [41]. NAC prevented excessive intracellular and extracellular ROS formation in mice with limb ischemia and enhanced the recovery of ischemic limb blood flow and function, in association with a selective increase in circulating endothelial progenitor cell (EPC) numbers (a group of cells critical for endothelial and vascular function) [42,43]. NAC treatment also attenuated C-reactive protein-induced ROS production in EPCs and apoptosis in vitro [44]. Animal studies also demonstrated that treating low-density lipoproteins (LDL) receptor deficient (LDLR KO) mice on a high-fat diet (HFD) with NAC in drinking water for 4 months significantly decreased ROS production and partially reversed the effects of hyperlipidemia on EPC populations [45]. Another animal study has revealed that NAC treatment effectively attenuated atherosclerosis progression following particulate matter (PM) exposure in LDLR KO mice with HFD, prevented excessive ROS generation, and reduced the levels of circulating oxidized LDL (ox-LDL) and inflammatory cytokines [46]. Preclinical animal studies have also shown that NAC normalized serum TNF-α level that was resistant to etanercept or infliximab, and improved HF in rats with cardiac injury [47,48,49]. A systematic review has demonstrated that NAC significantly decreased diabetes-associated cardiovascular complications including ischemia and non-ischemia cardiac damage through inhibition of oxidative stress in various animal models [23]. 

## 3. NAC and ROS

Excessive ROS plays a critical role in the development and progression of atherosclerosis. Many atherosclerogenic risk factors, including hypertension, diabetes, smoking, and dyslipidemia, increase ROS production, trigger inflammatory response, alter vascular function, and promote the growth of vascular smooth muscle [50,51,52]. NAC has been reported to prevent atherosclerosis formation in various animal models, as summarized in Table 4. Treatment of hypercholesterolemic rabbits with NAC significantly decreased the gelatinase expression, gelatinolytic activity, and matrix metalloproteinases (MMP)-9 expression in foam cells [53]. Similarly, it has been reported that treating atherosclerotic rabbits with NAC for 8 weeks significantly attenuated atherosclerotic formation, in association with a reduction in blood ox-LDL, MMP-9, MMP-2, and expression of MMP mRNA [54]. Using human THP-1 cells treated with phorbol 12-myristate 13-acetate for 48 h, followed by ox-LDL incubation for 4 days to induce foam cell formation, NAC treatment significantly reduced ROS production and ox-LDL uptake, leading to an inhibition of foam cell formation via a down-regulation of CD36 expression [55]. Another study reports that the treatment of apolipoprotein E knockout (ApoE KO) mice with NAC orally for 8 weeks significantly attenuated the progression of atherosclerosis, with decreased plaque collagen content and nitrotyrosine expression, probably via a reduction in oxidative stress [56]. In addition, aortic fatty streak plaque was effectively prevented in ApoE KO mice when treated with NAC via intraperitoneal injection for 8 weeks, with a reduction in aortic wall superoxide production [57]. A study shows that NAC treatment in drinking water for 12 weeks suppressed atherosclerotic development in ApoE KO mice with streptozotocin-induced type-1 diabetes, in association with improved GSH-dependent methylglyoxal elimination, decreased oxidative stress, and the restoration of phosphorylated Akt (p-Akt)/phosphorylated endothelial nitric-oxide synthase (p-eNOS) pathways in aortas [58]. 

Native LDL per se is not atherogenic, and ox-LDL is one of the key components in hyperlipidemic states and a potent source of ROS [45,64,65]. NAC could inhibit the in vitro oxidation of LDL and prevent the depletion of antioxidant vitamins [66]. One study has shown that NAC treatment also effectively attenuated the in vivo biotransformation of human native LDL to ox-LDL in a mouse model [60]. In a small study with 10 patients with CAD and hyperlipidemia, NAC treatment for 7 days significantly decreased the serum ox-LDL level, while there was no significant change in the serum ox-LDL level in patients with placebo [60]. These data suggest that NAC decreases ROS levels through multiple mechanisms, including an inhibition of the in vivo biotransformation of native LDL to ox-LDL.

## 4. NAC, Inflammation, and Macrophages

Inflammation is closely related to the development and progression of CVDs, especially atherosclerosis. Myeloid cells-mediated innate immune responses significantly contribute to chronic vascular inflammation [67]. It has been reported that treatment of human umbilical vein endothelial cells (HUVEC) with NAC effectively blocks the interleukin (IL)-4-induced expression of vascular cell adhesion molecule-1, which stimulates the adhesion of lymphocytes and monocytes to the surface of the vascular endothelium during the early phase of atherosclerosis development [68,69]. IL-6 is known to increase inflammation and the development of vascular diseases, including atherosclerosis. NAC treatment inhibits the production of IL-6 in acetoacetate-treated human U937 monocytes [70]. Lysophosphatidylcholine is produced from the hydrolysis of phosphatidylcholine by secretory phospholipase A2 (sPLA2) and has proinflammatory and proatherogenic effects on the vasculature [71]. NAC treatment significantly reduced TNF-α-induced expressions of group V sPLA2 (sPLA2-V) mRNA and protein in HUVEC [72]. Intraperitoneal injection NAC significantly attenuated balloon-induced neointimal formation in the carotid artery in rats via the inhibition of NF-κB activity in the medial smooth muscle cells [73]. Treatment of hyperlipidemic rabbits with a combination of the anti-inflammatory drug colchicine with fenofibrate or NAC for 7 weeks significantly reduced aortic atherosclerotic plaque. However, the atherosclerotic burden was significantly lower in the hyperlipidemic rabbits treated with a combination of colchicine with NAC compared with that of colchicine plus fenofibrate. Serum IL-6 levels were also significantly decreased in animals treated with colchicine plus NAC [63].

Macrophages are one of the important sources for inflammatory cytokines [74] and play a critical role in the pathogenesis of atherosclerosis [75]. An increase in macrophage polarization to proinflammatory macrophages (M1), or a decrease to anti-inflammatory macrophages (M2), increases the level of inflammation and promotes atherosclerotic progression [76]. Thus, the M1/M2 ratio is an important determinant for the direction of inflammatory response [77]. Macrophages are also important for the stability of atherosclerotic plaques [78]. The data from a study using aging LDLR^−/−^ mice showed that inflammatory markers (CRP, MCP-1, and IL-6) were significantly increased, while the anti-inflammatory cytokine IL-10 was significantly decreased in aging LDLR^−/−^ mice, in association with a significantly increased aortic ROS level and an increased M1/M2 ratio, largely due to decreased M2 population in the aorta. Further studies using bone marrow transplants with GFP-labeled bone marrow cells showed that the increased M1/M2 ratio in the aorta of aging LDLR^−/−^ mice was predominantly due to decreased M2 polarization, without a significant change in M1 polarization. NAC treatment effectively prevented changes in the expressions of pro-inflammatory and anti-inflammatory cytokines, the ROS level, and macrophage polarization in the aorta of aging LDLR^−/−^ mice. Interestingly, NAC treatment has no effect on the migration of monocytes from circulation into the aorta in aging LDLR^−/−^ mice or on M1 population in the aorta [59].

## 5. NAC and Atherosclerosis and CAD

Atherosclerosis and related CAD are a leading cause of mortality and morbidity in the world [79]. A multicenter clinical study, NAC in acute MI (NACIAM), with 112 patients, has shown that a combination of intravenous NAC treatment with a low dose of nitroglycerin (NTG) significantly shrinks the infarction size in patients with acute ST elevation MI undergoing primary percutaneous coronary intervention (PCI) [34]. Two more small studies (28 and 30 patients each) have reported that the combination of NAC with NTG and streptokinase reduced the levels of oxidative stress and plasma malondialdehyde (MDA), and improved left-ventricular function in patients with acute MI [35,36]. Similar results have been reported when NAC supplemented cold-blood cardioplegia [37] or when NAC was added to crystalloid cardioplegia in patients with CAD following a coronary artery bypass graft (CABG) [38]. NAC was also shown to potentiate the effects of NTG on the treatment of patients with unstable angina pectoris and other CAD patients [39,80]. A review of data from clinical studies has shown that NAC has cardioprotective effects in patients who had ischemic heart disease and underwent CABG and PCI [81]. However, a recent systematic review of 29 clinical trials with 2486 participants and a meta-analysis with 578 patients have demonstrated that NAC treatment does not significantly reduce major adverse events in patients undergoing cardiac surgery, including AMI and mortality [24,82] (Table 5).

Animal studies have shown that the intraperitoneal injection of NAC prevented nonthyroidal illness syndrome-related thyroid hormone derangement and preserved cardiac function in male rats with acute ischemic myocardial injury via the restoration of the redox balance [84]. Intravenous injection of NAC decreased oxidative stress, infarct area, and apoptosis in a rat cardiac ischemia-reperfusion injury model [85]. However, intracoronary administration of NAC in a pig model that simulated a catheter-based reperfusion model for the therapy of acute ST-elevated MI (STEMI) showed no significant effect on reducing the infarction size [86]. A recent study, using an aging LDLR^−/−^ mouse model with a regular diet, has demonstrated that NAC treatment significantly decreased aortic ROS levels and inflammatory cytokines in the serum and aortas of aging LDLR^−/−^ mice. The same study has also shown that early and adequate NAC treatment could effectively attenuate atherosclerosis progression in aging LDLR^−/−^ mice without extreme hyperlipidemia [59].

## 6. NAC and Lipid Metabolism

The disruption of the lipoprotein metabolism plays an important role in the development and progression of atherosclerosis [87]. Pretreatment with NAC significantly inhibited the differentiation of murine 3T3-L1 preadipocytes into adipocytes and decreased intracellular fat accumulation and the expressions of obesity-related proteins, including monoamine oxidase A, heat-shock protein 70, aminoacylase-1, and transketolase [88]. Similarly, NAC attenuates lipid accumulation and mitogen-activated protein kinases phosphorylation in murine embryonic fibroblasts during adipogenic differentiation [89]. Lipoprotein (Lp)(a) binds to apoprotein (a) and is considered an independent risk factor for premature atherosclerosis [90]. It has been shown that treating the serum from patients with a high concentration of Lp(a) with a high concentration of NAC (8 mg /mL or above) in vitro leads to dissociation of Lp(a) from apoprotein [91]. A small and yet significant reduction in Lp(a) concentration was observed in 12 subjects with a high serum Lp(a) level (87 mg/dL) following 6 weeks of NAC treatment [91]. However, another small clinical study of seven subjects with a median Lp(a) concentration of 14.3 mg/dL has demonstrated that NAC treatment for 6 weeks had no significant effect on plasma Lp(a) levels [91]. Similarly, no significant effect of NAC treatment on serum Lp(a) levels were found in 11 subjects with serum Lp(a) levels over 0.3 g/L [92]. An animal study has shown that treating LDLR KO mice on a HFD with NAC for 2 months or 4 months has no significant effect on the blood lipid profile, including triglycerides (TG), LDL, high-density lipoprotein (HDL), total cholesterol (TC), and non-HDL cholesterol [60]. Similarly, no significant effect of NAC treatment (250 mg/day, twice a day for 1 week) on the lipid profile was observed in human subjects with CAD and hyperlipidemia [60].

## 7. NAC and Homocysteine

An increased level of blood homocysteine is arguably considered a risk factor for atherosclerosis through increased oxidative stress, endothelial dysfunction, and thrombosis formation [93]. It has been reported that treating human subjects with NAC significantly reduced blood homocysteine levels by 45% over the placebo control [92]. The NAC treatment of patients with chronic renal failure led to a 16% reduction in plasma homocysteine levels [94]. Intravenous administration of NAC significantly decreased the level of plasma homocysteine in healthy subjects [95]. Data from controlled trials in middle-aged male subjects with unmedicated hyperlipidemia, with or without smoking, has shown that oral NAC treatment significantly reduced the level of total plasma homocysteine, regardless of lipid or smoking status [96]. However, Miner and colleagues have reported that treating cardiac transplant recipients with NAC had no significant impact on plasma homocysteine levels or brachial endothelial function [97].

## 8. Effects of NAC on Endothelial Cells

Endothelial dysfunction has been considered the first step of atherosclerosis development [98]. It was reported that treating endothelial cells from porcine pulmonary arteries with NAC significantly increased intracellular glutathione levels and partially prevented TNF-α-induced endothelial dysfunctions [99]. NAC also attenuated aortic endothelial damage in ApoE KO mice with streptozotocin-induced diabetes and HFD in association with increased levels of pAkt and -p-eNOS in aorta, as well as NO in serum [58]. Treatment of human aortic endothelial cells (HAEC) with NAC significantly attenuated TNF-α-induced ROS production and the DNA-binding activities of activator protein-1 and NF-κB, as well as p65 Ser276 phosphorylation [100]. Long-term treatment of endothelial cells (EC) from arterial segments of patients with severe CAD with NAC delayed senescence of diseased EC via the catalytic subunit of telomerase activation and transient telomere stabilization [101]. Intra-arterial infusion of NAC in healthy human subjects at a rate to achieve a blood concentration of 1 mM potentiated the effects of NTG on vasodilation and enhanced the biotransformation to an endothelium-derived relaxing factor equivalent nitrosothiol [102]. Intracoronary infusion of NAC in patients with or without coronary atherosclerosis significantly potentiated acetylcholine-induced coronary and femoral vasodilation and SNP-induced coronary vasodilation [103].

## 9. Mechanisms for the Actions of NAC on Atherosclerosis

The mechanisms for the effects of NAC on ROS generation, inflammation and atherosclerosis are very complex and have not been fully defined. Traditionally, NAC is considered to function as an antioxidant through a reduction in disulfide bonds or the scavenging of ROS or replenishing intracellular GSH stores [104]. However, in many settings and situations, the mechanisms of actions of NAC have remained unclear. Accumulating data has supported the concept that NAC is more like an anti-inflammatory agent with immunomodulatory properties, through its ability to attenuate the activation of oxidant-sensitive pathways, including the NF-κB and p38 mitogen-activated protein kinase (MAPK) signaling pathways, and subsequent reductions in pro-inflammatory cytokines such as TNF-α, IL-1β, and IL-6 [5,105,106]. 

**Figure 1:**
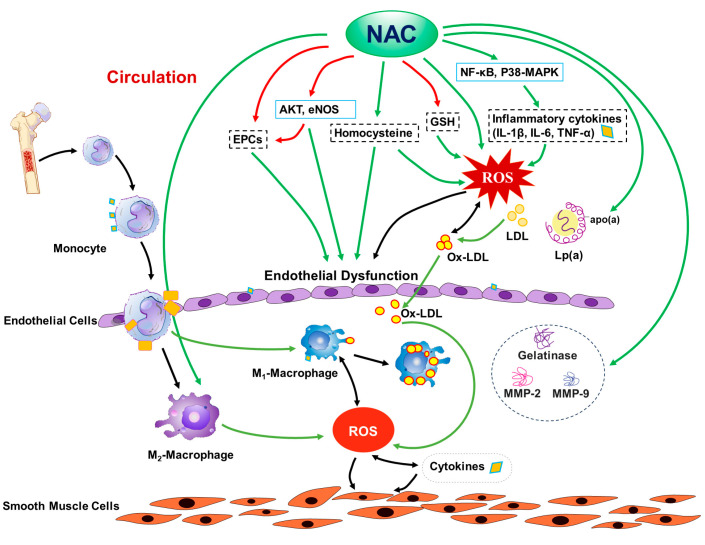
Potential mechanisms for the effect of N-acetylcysteine (NAC) on atherosclerosis. EPCs: endothelial progenitor cells; AKT: serine-threonine protein kinase; eNOS: endothelial nitric-oxide synthase; GSH: glutathione; IL: interleukin; LDL: low-density lipoprotein cholesterol; Lp(a): lipoprotein-a; apo(a): apoprotein-a; Ox-LDL: oxidized LDL; NF-κB: nuclear factor kappa-light-chain-enhancer of activated B cells; p38-MAPK: p38 mitogen-activated protein kinase; ROS: reactive oxygen species; TNF-α: tumor necrosis factor-alpha; M1: proinflammatory macrophages; M2: anti-inflammatory macrophages; MMP: matrix metalloproteinases; red arrows: increase; green arrows: decrease.

Due to the complex nature of atherosclerosis pathogenesis, the mechanisms for the effects of NAC on atherosclerosis are also complex, including (but not limited to) (1) modifications of lipid metabolism, (2) inhibition of the expressions of gelatinase, MMP-2, and MMP-9, (3) blocking the in vivo biotransformation of native LDL to ox-LDL, and directly suppressing ROS production from ox-LDL, (4) increasing intracellular glutathione levels, and thus protecting endothelial function, (5) attenuation of NF-κB and p38-MAPK signaling, thus decreasing inflammatory cytokine production, (6) the preservation of circulating endothelial cell progenitor cells, (7) reduction of homocysteine levels, (8) attenuation of endothelial senescence and damage, while enhancing endothelial function through multiple mechanisms, including activation of Akt signaling and eNOS, and (9) the preservation of M2 polarization in the hyperlipidemic condition, thus reducing inflammation and oxidative stress, as shown in Figure 1.

## 10. Other Effects of NAC and Mechanisms

Ambient fine PM exposure increases the risk of cardiovascular diseases, including atherosclerosis [107,108]. It has been reported that NAC treatment can significantly inhibit the motorcycle exhaust particulates-induced proliferation of rat aortic vascular smooth muscle cells (VSMC) through an extracellular signal-regulated kinase 1/2-activated cyclooxygenase-2 signaling pathway [109]. Another study demonstrated that NAC prevented a PM-mediated reduction in NO production in HAEC [110]. Animal studies have shown that NAC treatment effectively attenuated the PM exposure-induced production of intracellular ROS and inflammatory cytokines TNF-α, IL-1β, and IL-6, and preserved EPC populations in bone marrow and blood in mice with PM exposure [111,112,113].

Oral NAC treatment has been reported to significantly decrease blood pressure in human subjects with or without hyperlipidemia [96]. Intravenous NAC administration in mice promoted arterial thrombolysis that is resistant to recombinant tissue-type plasminogen activators, direct thrombin inhibitors, and antiplatelet treatments through targeting vWF cross-link platelets in the thrombi [114]. Co-administration of NAC and a GpIIb/IIIa inhibitor significantly enhanced the thrombolytic efficacy via accelerating thrombus dissolution and preventing re-thrombosis without increasing hemorrhagic stroke risk [114]. Interestingly, a study demonstrated that the combined application of vitamin C and desferrioxamine did not exhibit significant beneficial effects against myocardial ischemia-reperfusion injury in pigs [115]. However, another study, using isolated ventricular cardiomyocytes and cardiac fibroblasts from neonatal rats in a simulated ischemia/reperfusion model, showed that the combination of vitamin C, desferrioxamine, and NAC protected cardiac fibroblasts with enhanced survival and improved function [116]. It was also reported that the administration of NAC and melatonin effectively preserved the expression of miR-142-3p in cardiomyocytes in response to endothelin 1 and isoproterenol induced stress [117].

DiNAC is a disulfide dimer of NAC and functions as an immunomodulating drug with potent anti-atherosclerotic effects [118,119]. It has been reported that a 3-month treatment of hyperlipidemic rabbits with DiNAC decreased thoracic aortic atherosclerotic lesions by 50% [61]. Endothelial function is also significantly improved after 3 weeks of treatment with the same dose of DiNAC in hyperlipidemic rabbits [120]. Treatment of hyperlipidemic male subjects with either 100 or 500 mg/day DiNAC for 24 weeks substantially increased brachial artery diameters at rest and during hyperemia without affecting blood lipid levels [121]. The mechanisms for the actions of DiNAC are considered to work mainly through the immunomodulation and attenuation of TNF-α-induced reduction in NO production [61,121]. Another new agent, S-Nitroso-N-acetylcysteine (SNAC), is a derivative of NAC and a water-soluble S-nitrosothiol, and is capable of releasing NO directly for a variety of vasoactive activities [122]. Treating LDLR KO mice on a HFD with SNAC decreased murine aortic plaque by 55% through a decrease in constitutive NO synthase expression but had no effects on vasomotor function, with minor changes in the plasma lipid profile [62].

## 11. Tolerability and Potential Toxicity and Side Effects of NAC

NAC can be administered orally, intravenously, or by inhalation [5,123]. The terminal half-life of NAC is estimated to be between 6 h to 18 h after single- or multiple-dose administrations intravenously or orally, with oral bioavailability of about 4.0–10% [5,123,124]. Oral dosage forms of NAC include capsules, granulate and effervescent, fast-dissolving, and slow-release tablets. Following both single- and multiple-dose administration, the plasma concentration of NAC increases rapidly and reaches a peak at approximately 1–2 h [5,124]. The maximum plasma concentration is higher after multiple doses than after a single dose, as expected. Oral NAC treatment has been well-tolerated by patients with a daily dose as high as 2 g and is associated with very few side effects, with an excellent safety profile based on multiple clinical studies [13,20,23,125,126].

Although NAC has a well-established safety profile, its toxic/side effects may occur at extremely high doses or upon overdosing [5]. An in vitro study has demonstrated that the viability of human THP-1 cells is significantly decreased when exposed to NAC at a concentration of 6 mM or higher [55]. Animal studies have shown that symptoms of acute toxicity including ataxia, hypoactivity, labored respiration, cyanosis, loss of righting reflex, and convulsions could be apparent following a single intravenous administration of NAC at a dose of 1000 mg/kg in mice, 2445 mg/kg in rats, 1500 mg/kg in guinea pigs, 1200 mg/kg in rabbits, and 500 mg/kg in dogs [5]. Although the toxicity of an NAC overdose has not yet been defined in humans, in a case report, a paracetamol overdose patient was accidently given a dose of 100 g NAC (instead of 10 g) and in a short period of time developed hemolysis, thrombocytopenia, acute renal failure, and subsequently died [127]. Potential side/toxic effects of NAC include anaphylactoid reactions after parenteral administration (can be more severe or even cause death in patients with asthma); skin rash, urticaria, pruritus, acute flushing and erythema after intravenous administration; chest tightness, bronchoconstriction, bronchospasm, and increased airway obstruction after oral inhalation; and gastrointestinal symptoms after oral administration [5].

## 12. Other Antioxidants and Atherosclerosis

Many pre-clinical studies have shown that a variety of antioxidants could prevent atherosclerosis. However, the data on the cardiovascular outcomes from clinical studies in human subjects have been inconsistent and sometimes controversial [128]. In vitro and in vivo animal studies have demonstrated that vitamin A could prevent atherosclerosis through inhibiting VSMC proliferation and inflammation, increasing NO production via increased eNOS phosphorylation, modulating angiogenesis via vascular endothelial growth factor production, and downregulating the angiotensin II type 1 receptor [129]. Data from human studies have revealed that treatment with a combination of Vitamin A with Vitamin D could attenuate atherosclerosis through a reduction in serum IL-1β levels [130], downregulation of IL-17 and retinoid-related orphan receptor-c [131], upregulation of forkhead box protein-3 gene expression, and an increased number of regulatory T cells in patients with atherosclerosis [132]. The Carotene and Retinol Efficacy Trial with 52 subjects (2 diabetic and 2 on lipid-lowering medications) has shown that a 5-year supplementation with β-carotene and vitamin A leads to a small, nonsignificant elevation of serum TG during treatment and a decrease in serum TG level after discontinuing the treatment, without changes in serum HDL, LDL or TC levels [133]. Treating patients with vitamin E could prevent cell aging and LDL oxidation, reduce serum TG level, and increase serum HDL level in association with enhanced plasma apolipoprotein A1 concentration and decreased cholesteryl-ester transfer protein activity [134,135,136]. However, high dose of vitamin E usage could have adverse effects on the cardiovascular system mainly through impairing endothelium-dependent vasodilation and potential paradoxical prooxidant effect [129].

Another well-studied antioxidant, vitamin C, has also been reported to decrease the risk of CVDs by improving endothelial function via enhancing NO generation, preventing ox-LDL-induced cytotoxicity of VSMC, the expression of cell adhesion molecules, decreasing monocyte adhesion to the endothelium, and enhancing paraoxonase activity with vitamin E [129]. However, some studies have indicated an inverse relationship between vitamin C and CVDs, and raised concerns that the effects of vitamin C on CVDs in the studies using fruit and vegetables instead of pure vitamin C might be due to the atheroprotective effects of other nutrients in vitamin C-rich foods [129].

## 13. Unanswered Questions on NAC and Atherosclerosis and Challenges for Clinical Studies in Patients with Atherosclerosis

Abundant data supports the concept that NAC is a potent anti-inflammatory agent that attenuates the development and progression of atherosclerosis in pre-clinical animal studies. However, it is unclear if NAC could suppress the progression of atherosclerosis in humans. Many clinical studies were observational or included a small number of patients without randomization, with potential significant bias. A recent animal study showed that NAC treatment did not reverse the existing atherosclerotic lesions, and an extended period of treatment (6 months) was needed to show a beneficial effect of NAC on the progression of atherosclerosis in aging LDLR^−/−^ mice [70]. Thus, it could take years to demonstrate a significant difference in patients with NAC treatment, since atherosclerotic lesions usually progress slowly, and NAC treatment only prevents new lesion formation. Thus, it is very challenging to determine the effect of NAC treatment on the progression of atherosclerosis in patients without a prolonged period of follow up. In the same study, it was reported that long-term NAC treatment (6 months) had no effect on atherosclerosis progression in aging LDLR^−/−^ mice on HFD. No beneficial effect of NAC treatment on atherosclerosis progression was observed in aging LDLR^−/−^ mice on a normal diet when advanced atherosclerotic lesions were present [71]. This data strongly suggests that the timing and duration of NAC treatment, as well as the serum lipid level and disease stage, are critical factors for NAC therapeutic outcomes on atherosclerosis. NAC treatment was only effective when applied early, over a long period of time, with a reasonable control of the serum lipid level.

Inflammatory cytokines and chemokines are important for atherosclerosis development and progression. The Cardiovascular Inflammation Reduction Trial (CIRT) has shown that there were no cardiovascular benefits in patients with MI or type 2 diabetes or metabolic syndrome when there were no reductions in CRP, IL-1β, or IL-6 levels [137,138,139]. Consistent with these observations, the data from the CANTOS study has revealed that the patients with the greatest reductions in IL-6 and hs-CRP levels benefited the most from canakinumab treatment, with reduced adverse cardiovascular events [140]. Interestingly, a recent study revealed a positive association between the area of atherosclerotic lesions and serum levels of CRP and IL-6 in aging LDLR^−/−^ mice. Administration of NAC for six months effectively attenuated the levels of serum CRP and IL-6 in LDLR^−/−^ mice at the age of 15 months [71]. Thus, a well-designed clinical study is also needed to determine the effect of NAC on the serum levels of inflammatory cytokines, especially IL-6, hs-CRP, and cardiovascular mortality and morbidity in patients with atherosclerosis.

Recently, a new concept in polytherapy has been proposed, namely, to use multiple antioxidants together as a combined therapy with different antioxidants due to (1) ROS generation being a very complex process that involves a wide spectrum of sources of ROS and a variety of enzymes and/or singling pathways, and (2) antioxidants exhibiting very diverse characteristics chemically, biologically, and pharmacologically [141]. Thus, the combination therapy of different antioxidants may potentially generate significant synergistic effects on ROS suppression, leading to a much better clinical outcome. Certainly, NAC could be an important part of polytherapy in future pre-clinical and clinical studies to determine the efficacy of combination therapy for disease conditions associated with excessive ROS, including atherosclerosis.

Accumulating data has shown that many microRNAs (miRNA), including (but not limited to) miR-126-5p, miR-155, miR-146a, MiR-125a, miR-22, and miR143/145, may play an important role in the development and progression of atherosclerosis, as nicely summarized in a recent review [142]. These miRNAs are critically involved in the regulation of endothelial function, inflammation, macrophage polarization, lipid metabolism, the function of VSMCs, and plaque stability, as well as vascular calcification, thus significantly contributing to a variety of pathophysiological events at different stages of atherosclerosis. In addition, there are extensive interactions between miRNAs and inflammatory cytokines (including TNF-α, IL-1β, and IL-6) and chemokines (such as CCL5, CCL8, CXCL2, and CXCL4) [143,144]; thus, they are closely associated with ROS formation and oxidative stress. It has been reported that NAC treatment significantly decreased the levels of miR146a and NF-κB p65 signaling in rats [145] and reduced the expression of miR-21 and miR-29b induced by *C. parvum* treatment in mice [146]. These data suggest that NAC could attenuate atherosclerosis by targeting inflammatory miRNAs. Further studies are needed to define the effect of NAC on the expression profiles of miRNAs in atherosclerosis.

## 14. Conclusions

NAC and NAC derivatives (DiNAC and SNAC) seem to be promising therapeutic options to attenuate the development and progression of atherosclerosis through multiple mechanisms with a well-established safety profile. The significant beneficial effects of NAC on CVDs, including atherosclerosis, have been reported in many clinical studies. However, these studies mostly included a small number of study subjects with short-term treatment with NAC and short periods of follow up time. Atherosclerosis is a slow and progressive condition; thus, a large, randomized double-blinded clinical trial with long-term treatment and follow-up periods is needed to determine the effect of NAC on the development and progression of atherosclerosis and the clinical outcomes of patients with atherosclerosis.

## Figures and Tables

**Table 1:** Clinical studies with NAC in patients with peripheral vascular disease (PVD).

Patient Information	Intervention	Outcome	Ref.
Pts (6 M and 16 F, YOA: 18–75) with RP secondary to SSc	NAC i.v. starting with a 2 h loading dose of 150 mg/kg, then 15 mg/kg/h for 5 days	Both frequency and severity of RP attacks, active ulcers, and old challenge test mean recovery time decreased	[11]
Pts (7 M and 43 F; YOA: 35–67) with RP secondary to SSc	NAC i.v. 15 mg/kg/h for 5 h in every 14 days for about 3 years	Reduction of DU, RP attacks, and RP DU ulcer visual analog scale	[12]
Pts (4 M and 22 F, YOA: 25–68) with RP secondary to SSc	NAC i.v. 15 mg/kg/h for 5 h, every 2 weeks for 2 years	Increased global hands perfusion and decreased plasma adrenomedullin concentrations, frequency and severity of RP attacks	[13]
Pts (42 M, YOA: 32–58) with RP secondary to SSc	NAC oral 600 mg tid for 4 weeks	Decreased DU but no vasodilator effect on hands’ microcirculation	[14]
Pts (20 M and 4 F, YOA: 56–78) with stage 5 CKD during hemolysis	NAC i.v. 5 g in 5% glucose in a final volume of 50 mL during one hemodialysis session	Improved arterial vascular reactivity during reactive hyperemia with decreased reflective index	[8]
Pts (total 36, YOA: 56–76, without or with only minor signs of preoperative ischemia of the lower body) undergoing elective infrarenal AAA	NAC i.v. 150 mg/kg b.m. 30 min before infrarenal aortic clamping	Prevented elevation of plasma lipid peroxide, thromboxane, and prostacyclin levels after declamping with increased plasma GSH concentration for over 12 h	[10]

NAC: N-acetylcysteine; Pts: patients; M: male; F: female; YOA: years of age; RP: Raynaud’s phenomenon; SSc: systemic sclerosis; mg: milligram; kg: kilogram; h: hour; Ref: references; i.v.: intravenous infusion; DU: digital ulcer; tid: three times a day; AAA: abdominal aortic aneurysm; b.m.: body mass; b.w.: body weight; CKD: chronic kidney disease; GSH: glutathione.

**Table 2:** Clinical studies with NAC in atrial fibrillation (AF).

Patient Information	Intervention	Outcome	Ref.
Pts (total 150 M and F, YOA: 35–75) with elective CABG surgery using CPB	NAC i.v. 50 mg/kg for 30 min on days 1 and 2 after surgery	Reduced inflammation and incidence of POAF after CABG	[25]
Pts (231 M and 28 F, YOA: 53–73) with CABG or combined CABG and valve surgery	Carvedilol plus NAC i.v. 50 mg/kg for 1 h before surgery and at the same dose for 48 h after the procedure	Reduced oxidative stress and inflammation which were associated with POAF	[26]
Pts (231 M and 80 F, YOA: 54–72) with CABG or combined CABG and valve surgery	Carvedilol plus NAC i.v. 50 mg/kg/day for 1 h before surgery and at the same dose for 48 h after the procedure	Decreased POAF incidence and duration of hospitalization	[27]
Pts (44 M and 31 F, YOA: 56–76) with AF with CABG or valve surgery, or both	Amiodarone plus NAC i.v. 100 mg/kg 30 min and 25 mg/kg for 48 h	NAC plus amiodarone might facilitate converting POAF to SR, decrease the time to conversion, and lower the requirement of EC	[28]
Pts (91 M and 24 F, YOA: 25–78) with CABG or valve surgery, or both	NAC i.v. 50 mg/kg/day for 1 h before surgery and at the same dose for 48 h after the procedure	Decreased the incidence of postoperative AF	[29]
Pts (180 M and 60 F, YOA: 40–70) with CABG, with or without valve surgery	NAC orally 1200 mg bid starting 48 h before and up to 72 h after surgery	Had no significant effect on the incidence of POAF, in-hospital stay, and postoperative morbidity or mortality	[30]

NAC: N-acetylcysteine; Pts: patients; M: male; F: female; YOA: years of age; mg: milligram; kg: kilogram; h: hour; Ref: references; i.v.: intravenous infusion; AF: atrial fibrillation; CABG: coronary artery bypass graft; CPB: cardiopulmonary bypass; POAF: postoperative AF; SR: sinus rhythm; EC: electrical cardioversion.

**Table 3:** Clinical studies with NAC in coronary artery disease (CAD).

Patient Information	Intervention	Outcome	Ref.
Pts (47 M and 28 F, YOA: 50–78) with STEMI	NAC i.v. 29 g with NTG i.v. 7.2 mg over 2 days	Reduced infarct size in patients with STEMI undergoing PCI	[34]
Pts (total 28 M and F, YOA: ≤ 75) with AMI	NAC i.v. 15 g for over 24 h combined with NTG and streptokinase	Appeared to be safe for the treatment of evolving AMI and was associated with significantly less oxidative stress, a trend toward more rapid reperfusion, and better preservation of LV function	[35]
Pts (3 M and 19 F, YOA: 42–66) with AMI	NAC i.v. 15 g NAC for over 24 h combined with streptokinase	Diminished oxidative stress and improved LV function	[36]
Pts (19 M and 11 F, YOA: 55–61) with LVEF > 40% undergoing CABG	NAC i.v. 50 mg/kg b.w. with cold-blood cardioplegia	Minimized myocardial injury in the early hours after and during cardiac surgery	[37]
Pts (35 M, YOA: 59–63) with normal myocardial function undergone CABG	NAC i.v. 0.04 mol/L with Plegisol	Increased tissue capacity against oxidative stress and decreased inflammatory response	[38]
Pts (32 M and 14 F, YOA: 40–73) with severe unstable angina pectoris unresponsive to conventional treatment	NAC i.v. 5 g over 15 min after NTG and repeated every 6 h for 24 h.	Lowered incidence of AMI but increased symptomatic hypotension	[39]

NAC: N-acetylcysteine; Pts: patients; M: male; F: female; YOA: year of age; mg: milligram; kg: kilogram; h: hour; Ref: references; i.v.: intravenous infusion; STEMI: ST elevated myocardial infarction; NTG: nitroglycerin; PCI: percutaneous coronary intervention; AMI: acute myocardial infarction; LVEF: left ventricular ejection fraction; b.w.: body weight.

**Table 4:** Preclinical animal studies on NAC and atherosclerosis.

Animal Model	Intervention	Outcomes	Ref.
Aging mice (M, LDLR KO)	With ND or HFD for 24 mon, with NAC (1 mg/mL in drinking water) treatment for 3 or 6 mon.	Early and sufficient NAC treatment reduces inflammation and slows atherosclerosis progression in aging LDLR^−/−^ mice without HFD, maintaining M2 level with increased CD146.	[59]
Mice (M, ApoE KO)	With HFD and NAC (i.p. 20 mg/kg/day, 3 times a week for 8 weeks)	NAC may suppress atherosclerosis via reducing superoxide production.	[57]
Diabetic ApoE KO mice (M)	treptozotocin (i.p. for 5 days) and NAC (2 mmol/L in drinking water for 12 weeks)	NAC attenuates atherosclerosis in diabetic ApoE KO mice; correcting glutathione-dependent methylglyoxal elimination; reducing oxidative stress and restoring p-Akt/p-eNOS pathways.	[58]
Mice (F, ApoE KO) with chronic renal failure	NAC (200 mg/kg daily by mouth) or placebo for 8 weeks.	NAC can slow atheroma growth in uremia-related atherosclerosis in mice, likely by lowering oxidative stress.	[56]
Mice (M, WT, and LDLR KO)	WT: human native LDL (50 μg) or ox-LDL i.v. for 3 days with NAC pretreatment for 3 days (1 mg/mL in drinking water); LDLR KO: ND or HFD for 4 months with NAC (same dose) for 2 months.	NAC attenuates native LDL oxidation to ox-LDL and ROS generation from ox-LDL; NAC decreases atherosclerotic plaque formation in hyperlipidemic LDLR KO mice.	[60]
Mice (M, LDLR KO)	NAC (1 mg/mL in drinking water) with ND or HFD, plus PM exposure for either 1 week or 6 months	NAC prevents PM-induced atherosclerosis in association with reductions of ROS formation, ox-LDL, and inflammatory cytokines.	[46]
Rabbits (M, LDLR KO)	At 10 weeks of age, DiNAC (0.25 and 25 μmol/L in drinking water for 12 weeks.	DiNAC decreases atherosclerosis, possibly via immune regulations and antioxidant properties.	[61]
Mice (M, LDLR KO)	On ND or HFD, SNAC (0.51 μmol /kg i.p. for 15 days)	SNAC attenuates plaque development and improves endothelial cell function	[62]
New Zealand white rabbits (M)	Group1: HFD + colchicine (2 mg/kg/day) + fenofibrate (250 mg/kg/day; group 2: HFD + colchicine (2 mg/kg/day) plus NAC (15 mg/kg /day)	Colchicine reduces atherosclerosis, especially when combined with NAC. Colchicine blocks NLRP3 inflammasome, while NAC attenuates IL-6 signaling, reducing inflammation.	[63]

NAC: N-acetylcysteine; M: male; F: female; LDLR KO: Low-density lipoprotein receptor knockout; ApoE KO: Apolipoprotein-E knockout; Mon: months; ND: normal diet; HFD: high fat diet; M2: M2 macrophages; p-Akt: phosphorated Akt; eNOS: endothelial nitric-oxide synthase; DiNAC: N,N′-diacetyl-L-cystine; SNAC: S-nitroso-N-acetylcysteine; NLRP3: NOD-, LRR- and pyrin domain-containing protein 3; IL-6: interleukin 6.

**Table 5:** Clinical studies with NAC in other cardiac diseases.

Patient Information	Intervention	Outcome	Ref.
Pts (76 M and 58 F, YOA: 46–78) with end-stage renal failure	NAC orally 600 mg bid for 2 years	Reduced composite cardiovascular end points	[16]
Pts (29 M and 11 F, YOA: 58–70) with stable angina pectoris who underwent CABG	NAC i.v. 50 mg/kg/day for 3 days	Decreased pump-induced oxidoinflammatory response during CPB	[21]
Pts (31 M and 9 F, YOA: 57–75) with elective or urgent CABG	NAC i.v. 100 mg/kg into cardiopulmonary bypass prime followed by infusion at 20 mg/kg/h	Attenuated myocardial oxidative stress in the hearts of patients subjected to cardiopulmonary bypass and cardioplegic arrest	[22]
Pts (12 M and 2 F, YOA: 20–67) with severe chronic CHF	NAC i.v. 100 mg/kg body w.t. for over 30 min with 40–120 mg ISDN orally	Activated and potentiated the action of organic nitrates, improved CHF	[31]
Pts (14 M and 5 F, YOA: 12–63) with disease-free soft tissue sarcoma and doxorubicin-induced cardiomyopathy	NAC orally 5.5 g/m^2^ daily for 30 days	Had no effect in reversing longstanding doxorubicin-induced cardiomyopathy	[32]
Pts (YOA: >18) with a suspected, or confirmed diagnosis of Takotsubo Syndrome	NAC i.v. 10 g over 24 h	Will evaluate a therapeutic option in acute attacks of Takotsubo Syndrome	[83]

NAC: N-acetylcysteine; Pts: patients; M: male; F: female; YOA: years of age; mg: milligram; kg: kilogram; h: hour; Ref: references; i.v.: intravenous infusion; CABG: coronary artery bypass graft; CPB: cardiopulmonary bypass; CHF: congestive heart failure: ISDN: isosorbide dinitrate.

## Data Availability

Not applicable.
